# Using the five to fifteen-collateral informant questionnaire for retrospective assessment of childhood symptoms in adults with and without autism or ADHD

**DOI:** 10.1007/s00787-020-01600-w

**Published:** 2020-07-25

**Authors:** Tatja Hirvikoski, S. Lajic, J. Jokinen, E. Renhorn, A. Trillingsgaard, B. Kadesjö, C. Gillberg, J. Borg, The F. T. F. working group

**Affiliations:** 1 Pediatric Neuropsychiatry Unit, Department of Women’s and Children’s Health, Karolinska Institutet, Stockholm, Sweden; 2 Habilitation and Health, Region Stockholm, Stockholm, Sweden; 3 Center for Psychiatry Research, Region Stockholm, Stockholm, Sweden; 4 Department of Clinical Neuroscience, Karolinska Institutet, Stockholm, Sweden; 5 Pediatric Endocrinology Unit, Department of Women’s and Children’s Health, Karolinska InstitutetKarolinska University Hospital, Stockholm, Sweden; 6 Department of Psychology, University of Aarhus, Aarhus, Denmark; 7 Gillberg Neuropsychiatry Centre, Institute of Neuroscience and Physiology, Sahlgrenska Academy, University of Gothenburg, Gothenburg, Sweden; 8 Sahlgrenska University Hospital, Gothenburg, Sweden; 9 Center for Neurodevelopmental Disorders at Karolinska Institutet (KIND), CAP Research Center, Gävlegatan 22B, 11330 Stockholm, Sweden

**Keywords:** Neuropsychiatric, Attention-deficit/hyperactivity disorder, Parent ratings, Rating scale, Psychometric properties

## Abstract

Due to lack of previous studies, we aimed at evaluating the use of the Five to Fifteen (FTF) questionnaire in adults with neurodevelopmental disorders (NDD) and in controls without NDD. The NDD group consisted of adults with autism spectrum disorder ASD (*n* = 183) or attention-deficit/hyperactivity disorder (ADHD) (*n* = 174) without intellectual disability, recruited from a tertiary outpatient clinic. A web survey was used to collect data from general population adult control group without NDD (*n* = 738). The participants were retrospectively rated by their parents regarding childhood symptoms, using five to fifteen-collateral informant questionnaire (FTF-CIQ). Adults with NDD had higher FTF-CIQ domain and subdomain scores than controls, and displayed similar test profiles as children with corresponding diagnosis in previous studies. Based on the FTF-CIQ domain scores, 84.2% of the study participants (93% of the controls; 64% of the adults with NDD) were correctly classified in a logistic regression analysis. Likewise, Receiver Operating Characteristic (ROC) curve analysis on FTF-CIQ total sum score indicated that a cut-off value of 20.50 correctly classified 90% of the controls and 67% of the clinical cases, whilst a cut-off value of 30.50 correctly classified 84% of the controls and 77% of the clinical cases. The factor analysis revealed three underlying components: learning difficulties, cognitive and executive functions; social skills and emotional/behavioural symptoms; as well as motor and perceptual skills. Whilst not designed as a diagnostic instrument, the FTF-CIQ may be useful for providing information on childhood symptoms and associated difficulties in individuals assessed for NDD as adults.

**Electronic supplementary material:**

The online version of this article (10.1007/s00787-020-01600-w) contains supplementary material, which is available to authorized users.

## Introduction

The neurodevelopmental disorders (NDD:s) autism spectrum disorder (ASD) and attention-deficit/hyperactivity disorder (ADHD) are amongst the most common and fastest growing diagnostic groups in psychiatry. The increase in NDD diagnoses requires a corresponding increase in efforts and resources from clinical psychiatry to meet the needs for assessment and treatment of the patients.

A decade ago, the worldwide prevalence of ASD was estimated to about 1% [1], and for ADHD, 5–6% in children [2, 3] and 2.5–3.4% in adults [4, 5]. In the United States (US), biannual reports from the Centers for Disease Control and Prevention (CDC) have found an increase in ASD from 0.6% in 2002 to 1.69% in 2014 in 8-year-old children using a two-step surveillance system with multiple sources of information [6]. In a study of parent-reported diagnosis of ASD amongst school children, the prevalence had increased from 1.16% in 2007 to 2% in 2011–12 [7]. In a total population study of a community in South Korea, an ASD prevalence of 2.64% amongst 7- to 12-year-old children was found using a multi-informant screening procedure followed by a standardised clinical assessment [8]. Correspondingly, 2.5% of teenagers in the Stockholm County were reported to have a clinical diagnosis of ASD [9]. For ADHD, the age-specific prevalence in Scandinavia has increased 1.83‐fold in Finland, 2.95‐fold in Denmark and 7.21‐fold in Sweden during the period 1990–2007 according to a population-based study [10]. In Sweden, the 1-year prevalence of clinical ADHD diagnosis increased more than fourfold, and the annual prevalence doubled, between the years 2006 and 2011 [11]. In the US, population-based surveys have found that the estimated prevalence of diagnosed ADHD in children and adolescents increased from 6.1% in 1997–1998 to 10.2% in 2015–2016 [12]. In Taiwan, a national insurance record study indicated a 27-fold 1-year prevalence of clinical ADHD diagnosis from 1996–2005 and a 17-fold increase in incidence [13].

The rapid increase in prevalence of NDD:s has raised the question whether it reflects a true increase in prevalence, a capture of previously undiagnosed patients, or overdiagnosis. A number of factors contributing to the increase have been discussed including broadening of diagnostic criteria, increased awareness both amongst the public and amongst professionals, diagnostic substitution and access to healthcare. A comprehensive meta-analysis of 135 worldwide studies conducted over the last 3 decades found no evidence of a true increase of ADHD [14]. In addition, epidemiological studies of the underlying ASD and ADHD phenotypes found no increase over time in ASD- and ADHD-like traits on the extreme end during a 10-year period when the prevalence of ASD and ADHD diagnosis registered in the healthcare system increased several folds [15, 16]. Further support for lack of phenotypic increase of ASD comes from a familial study showing no increase in relative recurrence risks in family members regardless of increase in ASD diagnosis in the population [17]. Taken together, these results demonstrating stability in the ASD and ADHD phenotypes suggest that the increased prevalence in clinical diagnosis of ASD is more likely to reflect changes in environmental factors such as diagnostic practices, better access to medical services and/or current overdiagnosis, rather than a true increase in prevalence of NDD:s. Therefore, in clinical context, scientifically evaluated methods are of great importance for rigorous assessment and appropriate diagnostic processes.

Although the global trend is increased NDD prevalence, there are large national as well as regional differences suggesting underdiagnosis may still pose a problem. In the US, the ASD prevalence in children is 3% in New Jersey but only 1.3% in Arkansas. In Sweden, the number of children receiving pharmacological treatment for ADHD varies regionally between 2 and 14%. Clearly, these differences reflect a number of different factors including demographic variables, access to healthcare and awareness of NDD:s in society, and also differences in clinical practice in assessment of NDD:s. A recent meta-analysis of 15 Italian prevalence studies with varying rates of ADHD found that studies based on symptom reported a rate of 5.9% whereas those based on full clinical assessment of ADHD reported only 1.9% in paediatric populations [18], illustrating the profound effect of different diagnostic practices. A correct diagnosis, including assessment of associated difficulties and potential co-morbidity, is crucial for choice of interventions, and underdiagnosis poses as severe a problem as overdiagnosis in terms of lack of access to adequate treatment. Hence, the importance of comprehensive clinical assessment of NDD:s using validated instruments cannot be overstressed.

However, ASD and ADHD are clinically and aetiologically heterogeneous conditions, associated with, but not limited to, a variety of symptoms that can also be observed in other conditions. Some of the core symptoms such as concentration difficulties, social interaction difficulties and repetitive behaviour overlap partially with other conditions. Co-morbidity with depression, anxiety and OCD in adults with NDD:s has been estimated to over 50%, and overlap between different NDD:s is also common [19–24]. Thus, diagnosis of NDD:s requires a broader clinical assessment including developmental history and focusing not only at core symptoms [24–26].

In clinical settings, diagnosing NDD:s in the growing group of adult patients with IQ in the average range seeking assessment proves a challenge where emphasis on retrospective identification of neurodevelopmental symptoms is necessary since the vast majority of patients diagnosed with NDD:s in adulthood have a history of symptoms in childhood. However, in a subgroup of individuals, symptoms may have been less overt in childhood, and it is also common that adults seeking evaluation describe typical symptoms that are not currently included in the formal diagnostic DSM/ICD criteria [27] (e.g. executive dysfunctions [28], underperforming). Further, the number of symptom criteria fulfilled is not in concordance with the level of functional impairment experienced by the patients [29, 30]. Narrow assessments focusing on the criteria for a specific diagnosis without careful consideration of associated difficulties, co-morbidities and differential diagnoses may contribute to both over- and underdiagnosis. Hence, it is important to identify not only presence of formal ASD and ADHD core symptoms but also associated symptoms that belong to the developmental trajectories of ASD and ADHD.

In clinical assessment of NDD:s, self-rating questionnaires are commonly used for screening of symptoms. A number of questionnaires for self- and parent assessment of NDD symptoms exist, but few of these instruments are adapted for retrospective childhood assessment of adults. The Wender Utah Rating Scale (WURS) [31] is a questionnaire for adults’ self-rating of childhood symptoms of ADHD and the Social Communication Questionnaire (SCQ) [32] includes retrospective parent-rating of ASD symptoms, but none of these inventories systematically probe into other diagnoses. For a broad screening of NDD and psychiatric symptoms, the Autism–Tics, ADHD and other Comorbidities inventory (A-TAC) have been developed [33, 34]. Originally based on a screening questionnaire, A-TAC has been validated in children and adolescents cross-sectionally and longitudinally in population and community samples [26, 33, 34] administered as a telephone interview with parents. It has however not been validated for retrospective parent-rating of childhood symptoms in adults. A-TAC was developed using items from another instrument, the Five to Fifteen (FTF) questionnaire (freely available for download at www.5-15.org). The FTF was originally developed as a parent-rating questionnaire for assessment of ADHD, and its common co-morbid conditions and associated problems in children and adolescents aged 5–15 years [35]. It was constructed from a combination of clinical experience and symptom criteria in diagnostic manuals, and the factor structure was later examined in different child populations yielding somewhat different factor solutions [36–38]. The hitherto largest study of FTF included adolescents up to 17 years thus suggesting that FTF can be used for assessment in individuals older than 15 years [38]. However, studies examining retrospective use of FTF in older adolescents or adults still are lacking. The aim of the present study was to investigate the usefulness of the FTF questionnaire in retrospective parental assessment of childhood symptoms in the context of clinical assessment of NDD:s in adults; henceforth called five to fifteen-collateral informant questionnaire (FTF-CIQ). First, we examined the face validity of the FTF-CIQ scores obtained in adulthood by comparing the profiles of an adult clinical group and adult control group, to the profiles of comparable groups assessed during childhood and reported previously [35, 39]. Second, the questionnaire’s ability to discriminate between adult clinical and non-clinical groups was analysed. Finally, we extracted underlying factors of the FTF-CIQ in an adult population.

## Methods

The study was approved by the Regional Ethics Committee in Stockholm, Sweden, and has therefore been performed in accordance with the ethical standards laid down in the 1964 Declaration of Helsinki and its later amendments.

## The five to fifteen-collateral informant questionnaire (FTF-CIQ)

FTF-CIQ consists of the same 181 items (scored 0 = “Does not apply”; 1 = “Applies sometimes/to some extent”; 2 = “Definitely applies”) as the original FTF, divided in eight domains covering motor skills, executive functions, perception, memory, language, learning competencies, social skills and emotional/behavioural problems. The eight domains can be further divided into 22 subdomains, e.g. motor skills include subdomains gross and fine motor skills; executive functions include subdomains attention, hyperactivity–impulsivity, hypoactivity, as well as planning/organising, etc. (see Supplementary Table A for domains, subdomains and representative items).

Studies of the factor structure (parent- or teacher-reported profiles in children) have been conducted on the level of the 22 subdomains and have yielded different results; Bohlin and Janols found a two-factor solution of learning difficulties and socio-emotional problems [36], whereas Beltran-Ortiz et al. found one broad general development factor and three additional factors of (1) socio-emotional problems/control, (2) cognition/motor function/language, and (3) communication/school learning [37]. Bruce et al. reported six factors in a sample of children with ADHD: (1) cognitive skills, (2) motor/perception, (3) emotion/socialisation/behaviour, (4) attention, (5) literacy skills, and (6) activity control. The latter factor solution was similar to that in the hitherto largest study of FTF in children by Lambek and Trillingsgaard [38]. Other psychometric properties of the FTF domain and subdomain scores have been demonstrated in several studies finding acceptable to good internal consistency, test–retest reliability, and interrater agreement [35] and significant associations with relevant scores from other questionnaires [36] and performance-based measures [40, 41].

## Participants

### Adults with neurodevelopmental disorders (clinical sample)

The clinical sample was consecutively recruited from a tertiary clinic for assessment and treatment of adults with NDD:s in Stockholm, Sweden, during the years 2001–2013. The diagnostic assessment at the clinic was based on multiple sources of information: a clinical interview based on the DSM-IV criteria (American Psychiatric Association, 1994) was conducted in all cases. The patients also completed standardised self-rating questionnaires such as the Wender Utah Rating Scale, WURS [31] for the assessment of childhood ADHD symptoms. Collateral information was gathered by interviewing the participants’ significant others/family members to obtain a more complete diagnostic history of each individual. When available, additional information was obtained from records from child- and adolescent psychiatry, school health services, as well as adult psychiatry. The assessment also included neuropsychological testing with WAIS-R [42] or WAIS-III [43] and, in most cases, also other standardised tests, such as a continuous performance test [44, 45]. The diagnosis of ADHD or ASD was established after reaching consensus between the managing psychiatrist and clinical psychologist, both of whom had solid professional experience in the field of developmental disorders.

The instrument used in the assessment process differed somewhat during the time period for the study, depending on, e.g. availability of Swedish translations. However, a majority of patients (63% of individuals with ASD and 60% of individuals with ADHD) had a FTF-CIQ completed by a relative who knew them as a child. The FTF-CIQ was completed in almost all cases by the patients’ parents (in approximately two-thirds of cases mothers, although the sex of the parent was not recorded systematically).

The final clinical study group thus consisted of 174 adults with ADHD and 183 adults with ASD. In cases having both diagnoses, ASD was considered as the primary diagnosis and the individual with both ASD and ADHD (i.e. one-third of the ASD group) was thus classified to the ASD group. The demographic data for the study participants are summarised in Table 1.

**Table 1:** Sample characteristics at the time point for the retrospective assessment with the five to fifteen-collateral informant questionnaire in the three study groups

	General population control *n* = 719	ADHD *n* = 174	ASD *n* = 183	Statistics
Age at assessment/completed FTF-CIQ	*M* = 32.06 SD = 9.42 Min–max = 18–73	*M* = 33.02 SD = 9.98 Min–max = 18–63	*M* = 31.61 SD = 9.19 Min–max = 19–61	*F* (2, 1073) = 1.06, n.s.
Gender distribution	Males 398 (55.4%)/females 321 (44.6%)	Males 97 (55.7%)/females 77 (44.3%)	Males 124 (67.8%)/females 59 (32.2%)	*χ*^2^ (2) = 9.46, *Ф* = 0.094 *p* = 0.009
At least one additional DSM diagnosis	Not assessed	Yes *n* = 136 (78.2%)	Yes *n* = 148 (80.9%)	*χ*^2^ (1) = 0.40, n.s
Full-scale IQ (population norm * M* = 100, SD = 15)	Not assessed	*M* = 97.76 SD = 14.67 (*n* = 155)	*M* = 98.63 SD = 15.62 (*n* = 158)	*t* (311) = 0.51, n.s

Number in analysis given if deviant from the total number of cases in each group

*M* mean value, *SD* standard deviation, *n.s.* non-significant, *min* minimum, *max* maximum

### The control group

The control group was recruited using an invitation e-mail letter distributed to a web panel of adults in Sweden who had volunteered to participate in web surveys via a survey company (Bisnode/PFM). To increase the likelihood that the recipients were parents to at least one adult child, 25% of all e-mails were sent to individuals aged 45–50 years and 75% of e-mails were sent to individuals aged > 50 years. Only responders, who answered that they had at least one child > 18 years of age and with no NDD diagnoses, were eligible to fill out the FTF-CIQ. To reflect the distribution between mothers and fathers completing the FTF in both previous studies on children [38] and in the clinical sample (adults with NDD) in the current study, the gender distribution in our recipients was 75% females and 25% males. The final gender distribution amongst our control respondents was 23% fathers and 77% mothers. For other demographic data, see Table 1. To facilitate the retrospective completion of the FTF-CIQ, and to match to the instructions given to the parents of the clinical cases, a short text introduced the FTF-CIQ for the control group. The participants were instructed to “try to recall how their (now adult) offspring was when she/he was at school age”. Thus, the referred age range was more narrow than the original age range of the FTF (5–15 years), since the concept of “school age” in Swedish is generally interpreted as approximately 7–12 years.

### Statistical analyses

Missing data in single items in the clinical group were handled by calculating the domain and subdomain mean scores based on existing data. However, if there were more than single items missing in the subdomains, the mean scores were not calculated and these individuals were excluded either list-wise or pairwise depending on the analyses (see below).

Eighteen out of the 737 (2.44%) questionnaires completed by the parents from the control group were excluded due to age of the offspring (< 18 years). Due to the technical quality of the questionnaire (forced completion of each item before proceeding to the next one), there was only 0.1% missing data amongst the remaining/included 719 questionnaires.

To explore the face validity of using the FTF in the context of retrospective assessment of adults (i.e. the FTF-CIQ), the 5–15 profiles were drawn for the general population controls (*n* = 719), individuals with ADHD diagnosed as adults (*n* = 174) and individuals with ASD diagnosed as adults (*n* = 183), as well as comparable groups assessed during childhood reported in previous studies (controls *n* = 854; ADHD *n* = 57 and ASD *n* = 34) [39]. As a first proof of parents’ ability to rate childhood symptoms retrospectively for their adult offspring (face validity), the profiles were expected to resemble those obtained for children in previous studies.

The descriptive statistics of the FTF-CIQ domains and subdomains were expressed as mean values and standard deviations, and the between-group comparisons (control group; ADHD group; ASD group) were calculated using one-way ANOVA with post-hoc comparisons.

To analyse the classification ability of the FTF-CIQ, we conducted a logistic regression analysis and a receiver operating characteristics (ROC) curve analysis [46]. For these purposes, we combined the clinical NDD groups since the FTF was not designed for diagnostic assessment and is not expected to differ between different clinical groups, but may be expected to categorise clinical cases from non-clinical cases.

For the direct logistic regression analysis, the FTF-CIQ subscales (Motor skills, Executive functions, Perception, Memory, Language, Learning, Social skills, Emotional and behavioural problems) were entered simultaneously and the questionnaires’ ability to categorise the controls (specificity) and clinical cases (sensitivity; the clinical groups were combined) was calculated. The clinical cases missing data for one or more subscales (*n* = 26, i.e. 7.28%) were excluded list-wise. The examination of the residuals indicated that in total 7 cases were somewhat outside of the recommended limits (± 2.5), i.e. considered as outliers. However, since all cases had DFBeta values < 1 (in all subscales), and both leverage statistics and Cook’s distance indicated absence of any influential cases having an effect on the model, the data were deemed to be adequate for the analysis. Moreover, the Hosmer and Lemeshow Test indicated a good fit of the data in the model [*χ*^2^ (8) = 36.97, *p* < 0.001]. For the ROC curve, the total sum score of the FTF-CIQ was entered as the test variable whilst the dichotomous categorisation (clinical i.e. NDD/control) was used as the state variable. The area under the curve (AUC) was calculated with 95% confidence intervals.

To study the underlying factor structure of the FTF-CIQ (i.e. in the context of retrospective assessment of adults with NDD), we conducted a principal component analysis on the 22 FTF-CIQ subdomains for the sample of 719 controls. Missing data were handled by pairwise exclusion. Based on previous literature and the theoretical background, the factors were expected to correlate with each other why an oblimin rotation method with Kaiser Normalisation was chosen [46]. Both the Kaiser–Meyer–Olkin measure value (0.96) and highly significant Bartlett’s test (*p* < 0.001) indicated excellent sampling adequacy. Finally, the three groups’ (control, ADHD, ASD) sum scores were calculated for the obtained factors, and between-group comparisons were conducted using ANOVA. Effect sizes were expressed as partial eta squared ($\eta_{{\text{p}}}^{2}$) and interpreted as 0.01 = small effect size, 0.06 = moderate effect size, and 0.14 = large effect size [47].

## Results

### Descriptive statistics of adult patient and control groups and comparison to previous studies on children

Sample characteristics for the three groups (control group, ASD and ADHD, respectively) are depicted in Table 1. There were no between-group differences regarding age at assessment/when the FTF-CIQ was completed by parents, whilst the gender distribution differed slightly: the ASD group had proportionally more males compared to the controls or to the ADHD group. Psychiatric co-morbidity was common in both clinical groups. A clear majority of the clinical cases had completed IQ assessment as part of the clinical assessment procedure and both clinical groups had full scale IQ (FSIQ) within average range, well corresponding to population norm of *M* = 100, SD = 15.

**Fig. 1:**
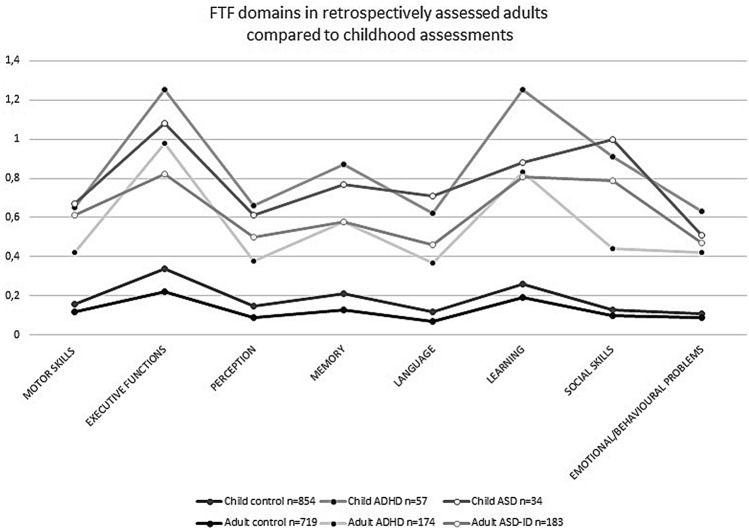
Comparison of mean scores in the FTF-CIQ domains in retrospectively assessed adults (intellectually able ASD and ADHD groups, respectively, as well as adult general population controls), to corresponding FTF scores assessed in children. The data on children have been published previously

Figure 1 shows the FTF-CIQ profiles for the adult groups included in current study compared to corresponding groups of FTF profiles regarding children from previous studies. The retrospective profiles for adults were similar to children’s profiles although the mean values were consequently somewhat lower in all three adult groups (controls, ADHD, ASD) compared to corresponding profiles in children.

Table 2 depicts the descriptive statistics and group comparisons in the FTF-CIQ domains and subdomains. Both NDD groups were reported to have more childhood symptoms in all domains compared to the controls, whilst the differences between the two NDD groups (ADHD *versus* ASD) were small. In one domain ADHD group was reported to have more difficulties than the ASD group (executive functions), whilst ASD group was reported to have more difficulties in three domains (motor skills, perception, and language). In four domains, there were no significant differences between the two NDD groups (memory, comprehension, learning, emotional and behavioural problems).

**Table 2:** Means and standard deviations of the FTF-CIQ domains and subdomains stratified according to the diagnostic status

FTF-CIQ domains and subdomains	Control (C) *n* = 719 *M* (SD)	ADHD *n* = 174	ASD *n* = 183	ANOVA statistics and between-group comparisons	Cronbachs * α* (for all participants combined * n* = 1076)^a^
Motor skills	*M* = 0.12 SD = 0.23	*M* = 0.42 SD = 0.46 (*n* = 167)	*M* = 0.61 SD = 0.52 (*n* = 175)	*F*(2, 1058) = 177.53 *p* < 0.001 C < ADHD < ASD	0.94
Gross motor skills	*M* = 0.14 SD = 0.29	*M* = 0.46 SD = 0.56 (*n* = 167)	*M* = 0.77 SD = 0.64 (*n* = 175)	*F*(2, 1058) = 175.68 *p* < 0.001 C < ADHD < ASD	0.91
Fine motor skills	*M* = 0.10 SD = 0.23	*M* = 0.39 SD = 0.47 (*n* = 166)	*M* = 0.50 SD = 0.54 (*n* = 175)	*F*(2, 1057) = 120.74 *p* < 0.001 C < ADHD < ASD	0.90
Executive functions	*M* = 0.23 SD = 0.34	*M* = 0.98 SD = 0.51 (*n* = 173)	*M* = 0.82 SD = 0.51 (*n* = 182)	*F*(2, 1071) = 338.54 *p* < 0.001 C < ASD < ADHD	0.97
Attention	*M* = 0.26 SD = 0.41	*M* = 1.17 SD = 0.61 (*n* = 173)	*M* = 1.01 SD = 0.62 (*n* = 182)	*F*(2, 1071) = 352.51 *p* < 0.001 C < ASD < ADHD	0.95
Hyperactive/impulsive	*M* = 0.18 SD = 0.33	*M* = 0.74 SD = 0.62 (*n* = 169)	*M* = 0.46 SD = 0.54 (*n* = 178)	*F*(2, 1063) = 130.04 *p* < 0.001 C < ASD < ADHD	0.92
Hypoactive	*M* = 0.22 SD = 0.38	*M* = 0.98 SD = 0.60 (*n* = 168)	*M* = 1.06 SD = 0.66 (*n* = 177)	*F*(2, 1061) = 326.30 *p* < 0.001 C < ASD = ADHD	0.88
Planning/organising	*M* = 0.26 SD = 0.44	*M* = 1.11 SD = 0.68 (*n* = 169)	*M* = 0.97 SD = 0.72 (*n* = 175)	*F*(2, 1060) = 251.55 *p* < 0.001 C < ASD < ADHD	0.85
Perception	*M* = 0.09 SD = 0.19	*M* = 0.38 SD = 0.40 (*n* = 173)	*M* = 0.50 SD = 0.49 (*n* = 182)	*F*(2, 1071) = 166.98 *p* < 0.001 C < ADHD < ASD	0.91
Relation in space	*M* = 0.09 SD = 0.22	*M* = 0.36 SD = 0.51 (*n* = 166)	*M* = 0.48 SD = 0.58 (*n* = 171)	*F*(2, 1053) = 103.84 *p* < 0.001 C < ADHD > ASD	0.80
Time concepts	*M* = 0.12 SD = 0.27	*M* = 0.49 SD = 0.59 (*n* = 173)	*M* = 0.49 SD = 0.62 (*n* = 174)	*F*(2, 1071) = 98.22 *p* < 0.001 C < ADHD = ASD	0.84
Body perception	*M* = 0.11 SD = 0.25	*M* = 0.45 SD = 0.49 (*n* = 171)	*M* = 0.64 SD = 0.62 (*n* = 180)	*F*(2, 1067) = 165.29 *p* < 0.001 C < ADHD < ASD	0.79
Visual perception	*M* = 0.06 SD = 0.18	*M* = 0.22 SD = 0.45 (*n* = 170)	*M* = 0.30 SD = 0.49 (*n* = 178)	*F*(2, 1064) = 57.51 *p* < 0.001 C < ADHD = ASD	0.76
Memory	*M* = 0.13 SD = 0.26	*M* = 0.58 SD = 0.47 (*n* = 165)	*M* = 0.58 SD = 0.51 (*n* = 176)	*F*(2, 1057) = 207.84 *p* < 0.001 C < ADHD = ASD	0.90
Comprehension	*M* = 0.10 SD = 0.27	*M* = 0.52 SD = 0.58 (*n* = 165)	*M* = 0.55 SD = 0.60 (*n* = 176)	*F*(2, 1057) = 141.63 *p* < 0.001 C < ADHD = ASD	0.89
Language	*M* = 0.08 SD = 0.18	*M* = 0.37 SD = 0.38 (*n* = 173)	*M* = 0.46 SD = 0.46 (*n* = 182)	*F*(2, 1071) = 175.51 *p* < 0.001 C < ADHD < ASD	0.91
Expressive language skills	*M* = 0.06 SD = 0.16	*M* = 0.26 SD = 0.36 (*n* = 170)	*M* = 0.37 SD = 0.44 (*n* = 180)	*F*(2, 1066) = 111.47 *p* < 0.001 C < ADHD < ASD	0.90
Communication	*M* = 0.09 SD = 0.29	*M* = 0.57 SD = 0.57 (*n* = 164)	*M* = 0.72 SD = 0.70 (*n* = 174)	*F*(2, 1054) = 192.00 *p* < 0.001 C < ADHD < ASD	0.86
Learning	*M* = 0.19 SD = 0.33	*M* = 0.83 SD = 0.54 (*n* = 170)	*M* = 0.81 SD = 0.56 (*n* = 180)	*F*(2, 1066) = 268.05 *p* < 0.001 C < ADHD = ASD	0.96
Reading/writing	*M* = 0.21 SD = 0.39	*M* = 0.68 SD = 0.67 (*n* = 157)	*M* = 0.75 SD = 0.67 (*n* = 164)	*F*(2, 1037) = 116.97 *p* < 0.001 C < ADHD = ASD	0.93
Math	*M* = 0.19 SD = 0.43	*M* = 0.79 SD = 0.74 (*n* = 154)	*M* = 0.79 SD = 0.75 (*n* = 161)	*F*(2, 1031) = 130.23 *p* < 0.001 C < ADHD = ASD	0.95
General learning	*M* = 0.10 SD = 0.26	*M* = 0.60 SD = 0.60 (*n* = 168)	*M* = 0.70 SD = 0.63 (*n* = 171)	*F*(2, 1055) = 211.00 *p* < 0.001 C < ADHD = ASD	0.86
Coping in learning	*M* = 0.21 SD = 0.37	*M* = 1.05 SD = 0.56 (*n* = 166)	*M* = 0.92 SD = 0.60 (*n* = 173)	*F*(2, 1055) = 342.30 *p* < 0.001 C < ASD < ADHD	0.94
Social skills	*M* = 0.10 SD = 0.24	*M* = 0.44 SD = 0.40 (*n* = 169)	*M* = 0.79 SD = 0.49 (*n* = 179)	*F*(2, 1064) = 360.90 *p* < 0.001 C < ADHD < ASD	0.96
Emotional/behavioural problems	*M* = 0.09 SD = 0.18	*M* = 0.42 SD = 0.37 (*n* = 172)	*M* = 0.47 SD = 0.37 (*n* = 182)	*F*(2, 1070) = 229.35 *p* < 0.001 C < ADHD = ASD	0.94
Internalising	*M* = 0.12 SD = 0.22	*M* = 0.49 SD = 0.45 (*n* = 168)	*M* = 0.66 SD = 0.50 (*n* = 177)	*F*(2, 1061) = 238.46 *p* < 0.001 C < ADHD < ASD	0.90
Externalising	*M* = 0.09 SD = 0.23	*M* = 0.50 SD = 0.47 (*n* = 167)	*M* = 0.39 SD = 0.40 (*n* = 174)	*F*(2, 1057) = 157.00 *p* < 0.001 C < ASD < ADHD	0.91
Tics and obsessive–compulsive	*M* = 0.04 SD = 0.15	*M* = 0.23 SD = 0.35 (*n* = 172)	*M* = 0.30 SD = 0.41 (*n* = 180)	*F*(2, 1068) = 95.78 *p* < 0.001 C < ADHD < ASD	0.81

Missing domain scores among the ADHD and ASD groups were handled with pairwise exclusion and the exact numbers of individuals in each analysis are given if deviating from the full group size

*M* mean value, *SD* standard deviation, *α* Cronbach’s alpha

^a^In the analyses of Cronbach’s alpha, like in the group comparisons, the *n* varied depending on the missing data among the clinical (ADHD and ASD) groups in certain variables, as indicated by the *n* depicted in the descriptive statistics

### Classification accuracy of controls and clinical cases

After exclusion of the cases missing one or more FTF-CIQ domain scores, the logistic regression analysis included *n* = 719 controls and *n* = 331 individuals with ADHD and/or ASD (the clinical group). The model included the eight FTF-CIQ domain as predictors of group status, and the test of the full model was statistically significant *χ*^2^ (8, *n* = 1050) = 484.35, *p* < 0.001, indicating that the model reliably distinguished between clinical and control cases. The variance in the group status accounted for by the model varied between small (Cox and Snell *R*^2^ = 0.37) and medium (Nagelkerke *R*^2^ = 0.52) depending on the used statistics.

Classification was excellent regarding specificity but poorer regarding sensitivity; 93% of the controls and 65% of clinical cases were correctly classified, consequential overall classification percentage being 83% (Table 3). Table 4 shows the Wald statistics, and the exponentiations of the beta coefficients (i.e. odds ratios) with 95% confidence intervals. The Executive functions subscale and the Social skills subscale contributed most to the logistic regression model; one unit change in the EF subscale resulted in 4.49 higher likelihood of being categorised in the clinical group whilst the corresponding odds ratio (exp Beta) for Social skills subscale was 5.65. The Memory and Language domains did not contribute to the regression model at the level of statistical significance or trend.

**Table 3:** Classification table from the logistic regression analyses predicting group status (control/clinical, i.e. ASD and/or ADHD) based on the FTF-CIQ domains

Observed	Predicted		Classification accuracy (%)
	Control	Clinical (NDD group)	
Control (*n* = 719)	669	50	93.0
Clinical (*n* = 331)	116	215	65.0
Overall percentage correct			84.2

*NDD* neurodevelopmental disorder, i.e. ADHD and/or ASD

**Table 4:** The bivariate Pearson’s correlation coefficients between the FTF-CIQ domains (calculated for controls and NDD groups combined) and the contribution of each domain to the logistic regression model

FTF domains	Motor	EF	Perception	Memory	Language	Learning	Social	Emotional	Wald (*df* = 1)	Sig	Exp.(*β*)	95% CI for EXP (*β*)	
												Lower	Upper
Motor skills	1								3.59	0.058	2.09	0.97	4.46
Executive functions	0.660**	1							19.27	0.000	4.49	2.30	8.78
Perception	0.783**	0.715**	1						7.40	0.007	0.21	0.07	0.64
Memory	0.631**	0.794**	0.798**	1					0.96	0.328	1.60	0.62	4.14
Language	0.648**	0.692**	0.771**	0.782**	1				0.00	0.985	0.99	0.38	2.61
Learning	0.679**	0.820**	0.738**	0.838**	0.777**	1			3.32	0.068	1.91	0.95	3.85
Social skills	0.674**	0.708**	0.716**	0.661**	0.691**	0.707**	1		20.23	0.000	5.65	2.66	12.02
Emotional/behavioural	0.571**	0.724**	0.670**	0.664**	0.647**	0.682**	0.795**	1	4.84	0.028	3.07	1.13	8.34

*CI* confidence interval, *Exp.(β)* exponentiation of the *β* coefficient odds ratio, *df* degrees of freedom, *Sig.* significance value

*p < 0.05; **p < 0.01; ***p < 0.001

**Fig. 2:**
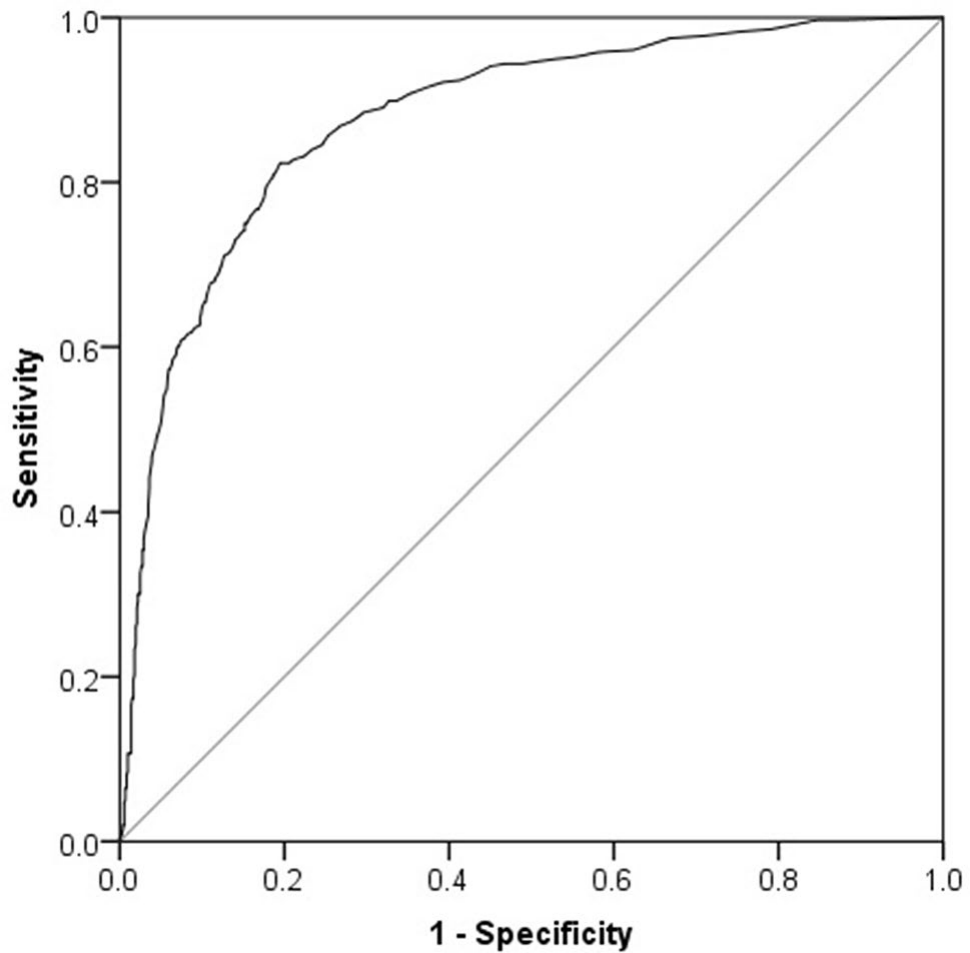
The ROC curve indicated a good classification ability of the FTF-CIQ total sum score. A cut-off value of 20.50 correctly classified 90% of the controls and 67% of the clinical cases (ADHD and ASD combined)

The ROC analysis (Fig. 2) using the FTF-CIQ sum score (sum of all item scores) also indicated a good classification ability. The AUC was 0.88 (95% CI 0.86–0.90), *p* < 0.001. A cut-off value of 20.50 indicated that 90% of the controls and 67% of the clinical cases (ADHD and ASD combined) were correctly classified, whilst a cut-off value of 30.50 classified 84% of the controls and 77% of the clinical cases.

### Factor analysis

Three factors with eigenvalues > 1 were extracted from the principal component analysis including the data from the controls (*n* = 719). Table 5 depicts the factor loadings and factor correlations. Before rotation, the model explained 68.37% of the total variance (since we conducted an oblique rotation, the components were allowed to covary and thus the sums of squared loadings cannot be added to obtain a total variance after the rotation). The three factors had medium strong correlations with each other, further supporting the choice of oblique rather than orthogonal rotation method.

**Table 5:** The factor loadings and factor correlations for the three extracted factors (components) from the principal components analysis with oblimin rotation, calculated on the scores of the control group (*n* = 719)

	Extracted components			
	Learning, cognitive and executive functions	Social skills and emotional/behavioural symptoms	Motor and perceptual skills	Cronbach’s alpha coefficient (*n* = 719 controls)
Coping in learning	0.940			0.92
Attention	0.852			0.92
Planning/organising	0.844			0.80
Reading/writing	0.810			0.92
Math	0.805			0.94
Memory	0.769			0.84
Hypoactive	0.681			0.81
Comprehension	0.635			0.87
Hyperactive/impulsive	0.634			0.90
Time concepts	0.596			0.75
General learning	0.581			0.82
Communication	0.535			0.84
Externalising		0.783		0.91
Tics and obsessive–compulsive		0.713		0.80
Social skills		0.661		0.96
Internalising		0.632		0.86
Gross motor skills			0.768	0.85
Fine motor skills			0.701	0.86
Visual perception			0.675	0.69
Relation in space			0.668	0.71
Body perception			0.639	0.73
Expressive language	(0.450)		0.481	0.86
*Factor correlations*				
Factor 1	1			0.95
Factor 2	0.530	1		0.88
Factor 3	0.636	0.428	1	0.87

The factor loadings < 0.40 were suppressed

The factor loadings < 0.40 were suppressed and the inspection of the pattern matrix showed strong factor loading (all but one > 0.50) for all subdomains and only one subdomain loading on two factors: expressive language skills had a factor loading > 0.45 for both components 1 and 3. This may be explained by the items tapping both cognitive aspects of expressive language such as finding words and expressing him/herself with grammatically correct sentences (that may load on factor 1) and motor aspects of expressive language such as speech sounds and pronunciation of difficult words (probably loading on factor 3).

In summary, the factors were labelled (1) learning difficulties, cognitive and executive functions; (2) social skills and emotional/behavioural symptoms; (3) motor and perceptual skills. The comparison of the three groups’ (control, ADHD, ASD) sum scores on the three obtained factors, as well as between-group comparisons and ANOVA statistics are depicted in Table 6. The effect of the diagnostic status was large in all three factors. However, the large difference was between controls compared to both groups with NDD, whilst the results were more overlapping between the ADHD and the ASD groups, as indicated by the mean values and the standard deviations (Table 6).

**Table 6:** Comparison of the two patient groups’ (ADHD group and ASD group, respectively) scores to the control group’s scores, on the three factors obtained from the factor analysis

Factor	Control (C) *n* = 719	ADHD *n* = 174	ASD *n* = 183	ANOVA statistics and between-group comparisons
1. Learning, cognitive and executive functions	*M* = 2.06 SD = 3.34	*M* = 9.16 SD = 5.32 (*n* = 151)	*M* = 8.85 SD = 5.92 (*n* = 150)	*F*(2, 1017) = 297.80 *p* < 0.001, * η*^2^ = 0.37 C < ASD = ADHD
2. Social skills and emotional/behavioural symptoms	*M* = 0.35 SD = 0.73	*M* = 1.65 SD = 1.39 (*n* = 165)	*M* = 2.12 SD = 1.39 (*n* = 173)	*F*(2, 1054) = 287.50 *p* < 0.001, * η*^2^ = 0.35 C < ADHD < ASD
3. Motor and perceptual skills	*M* = 0.56 SD = 1.06	*M* = 2.13 SD = 2.17 (*n* = 162)	*M* = 3.00 SD = 2.56 (*n* = 166)	*F*(2, 1044) = 191.20 *p* < 0.001, *η*^2^ = 0.27 C < ADHD < ASD

The number of individuals in the ADHD and ASD groups varied due to missing data, and therefore, the exact number in each analyses is indicated

*M* mean value, *SD* standard deviation, *n* = number; *η*^2^ partial eta squared effect size

## Discussion

In this study of retrospective assessment of childhood symptoms of NDD:s in adults, we found that the FTF-CIQ profiles for the adult ASD, ADHD and general population control groups had high similarity to the profiles for the corresponding FTF profiles of children in prior studies. This result indicates good face validity of the FTF-CIQ for retrospective parent-rating of childhood NDD symptoms in adults.

The retrospective mean values for our three adult groups were consistently lower compared to the corresponding values for children, which is expected as individuals diagnosed with NDD:s in adult age differ in their cognitive and symptom profiles from those diagnosed in childhood [48]. The phenotypic manifestation of ASD and ADHD varies throughout development and into adulthood, and it is not known to what extent these variations represent an age-related change in the phenotype, or capture phenotypical differences in patient subgroups [49, 50]. ASD and ADHD have heterogeneous geno- and phenotypes with subgroups with different developmental trajectories existing in both diagnostic groups. Individuals receiving a first diagnosis of ASD or ADHD in adult age typically have intellectual abilities in the normal range, facilitating their ability to “camouflage” their difficulties and develop partially compensatory strategies “masking” symptoms [50, 51]. Compared to early diagnosed individuals, they may differ also with regard to quality and quantity of symptom presentation, access to assessment services and level of support from social environments.

The factor structure of FTF has been previously investigated only regarding children and adolescents. A study of children from a Swedish population register (*n* = 1500) found a two-factor solution of learning difficulties and socio-emotional problems [36], whereas a population-based study of children in Chile (*n* = 322) resulted in one broad general development factor and three additional factors representing socio-emotional problems/control, cognition/motor function/language, and communication/school learning [37]. In a Swedish clinical sample of children with ADHD (*n* = 76), a similar factor structure was identified as in a study of Danish children and adolescents rated by both parents (*n* = 4258) and teachers (*n* = 1298); a factor solution of six domains including cognitive skills, motor/perception, emotion/socialisation/behaviour, attention, literacy skills and activity control [38]. In our study, a three-factor solution was revealed consisting of (1) learning difficulties/cognitive and executive functions, (2) social skills/emotional/behavioural symptoms, and (3) motor and perceptual skills. The first two factors comprise subdomains corresponding well to core symptoms and associated problems of ADHD and ASD, respectively. The third factor, motor and perceptual skills, is related to symptoms that have long been under-researched. For ASD, hyper- and hyposensitivity to sensory stimuli were included in the diagnostic criteria only in DSM-5 [52]. Sensory sensitivity has a high prevalence both amongst individuals with ASD and individuals with autistic traits [53]. Many individuals with ASD perceive perceptual symptoms as more impairing than the socio-communicative symptoms [54], and sensory sensitivity have also been associated with sleep disturbances [55]. Anomalies in sensory perception are however not unique to ASD; in ADHD atypical sensory profiles and hypersensitivity are common [56, 57], also irrespective of co-occurring autistic symptoms [58]. Motor skill impairments are common in both ASD and ADHD, with higher prevalence in children with ASD [59]. Identification of childhood motor impairments is of particular importance, since poor motor skills are highly correlated to risk of being bullied in school [60], and thus related to social difficulties as well.

In the retrospective childhood data on adults, we observed between-group differences (controls versus clinical cases) comparable to previous studies on children [38, 39]. Also in line with studies of the FTF on children, the differences in the FTF-CIQ domains and subdomains between the two clinical groups (ASD and ADHD) were relatively small, and the two clinical groups were combined in the logistic regression and ROC analyses. Using the FTF-CIQ domain scores as predictors in a logistic regression analysis, 84% of the total sample could be correctly classified (93% of the controls, and 65% of the clinical groups). Likewise, in the ROC analysis, a cut-off value of 20.50 in the summed score for all items correctly classified 90% of the controls and 67% of the clinical cases (ADHD and ASD combined). Using a cut-off value of 30.50 of the total summed score, 84% of the controls and 77% of the clinical cases were correctly classified. Thus, the FTF-CIQ is clearly not a diagnostic instrument due to the poor ability to discriminate between different clinical groups (ASD versus ADHD) and due to the relatively poor sensitivity. However, it should be noted that the FTF was not developed for diagnostic purposes but for broad clinical assessment and description of NDD symptoms and associated difficulties and relative strengths in children. Our results indicate that the FTF-CIQ can be used for gathering additional collaborative information also in the context of retrospective assessment of adults. In clinical practise, adult diagnoses of NDD may be missed due to co-morbid psychiatric conditions partially overlapping with and obscuring ASD and ADHD symptoms. It is well known that adults with NDD:s have difficulties recalling childhood symptoms [24], often underestimating symptom severity compared to parent reports and failing to identify the connection between symptoms and function disability [30, 61, 62]. Although report bias exist in both patients and parents, it is generally recommended that retrospective information is collected from several informants in clinical practice [63, 64]. For this purpose, FTF-CIQ can be used. To our knowledge, the only alternative instrument encompassing a broad, systematic assessment of both ASD, ADHD and associated NDD and psychiatric symptoms is the A-TAC which has been validated as a telephone interview. However, it has not been validated for retrospective assessment of childhood symptoms in adults.

The global increase in prevalence of ASD and ADHD has been extensively debated in recent years, as has the increase in the number of individuals treated for NDD:s. The prescription of ADHD medication has increased between two- and sevenfold in both Europe and the US [16]; for example, ninefold for children and adults in the UK from 2000 to 2015 [65] and in Sweden, medication more than doubled from 2006 to 2009 for both children and adults with the most prominent increase for adults [66]. The number of young adults receiving healthcare for ADHD and/or ASD has more than doubled from the year 2011 to 2016 [67]. For ADHD, the prevalence of diagnosis and subsequent pharmacological treatment is expected to increase even further with the application of the DSM-5 criteria that allow children with sub-threshold symptoms and no functional impairment to meet the diagnostic criteria for ADHD later in life [68]. Simultaneously, the large regional differences in prevalence suggest that underdiagnosis still occurs. Both over- and underdiagnosis have profound effects for the patients and their families in terms of inadequate, or lack of, treatment. For ADHD, wide prevalence ranges have been reported in older as well as recent studies and the prevalence of both ADHD diagnosis and medication has increased more in the US than in Europe [12, 69, 70]. Some factors influencing the prevalence have been identified, such as lower income households having lower prevalence [4]. In the US, CDC studies have demonstrated a relationship between prevalence and accessibility to specialised healthcare centres performing assessments, with higher prevalence in areas with better access to services. In Sweden, the National Board of Health and Welfare recently published a report concluding that socioeconomic factors do not contribute to the varying geographic prescription rate of ADHD medication, but access to healthcare and differences in clinical treatment practise do. Similarly, it is likely that differences in clinical diagnostic assessment practise play a role in the varying prevalence rates. In a meta-analysis of 86 studies of children and adolescents (*N* = 163,688 individuals) and 11 studies of adults (*N* = 14,112 individuals) of ADHD prevalence, almost all (97–99%) children who eventually met the criteria for ADHD, exhibited symptoms and impairment by the age of 10 [3]. Hence, validated instruments for assessment of childhood symptoms in adults seeking assessment for NDD are required.

The results of our study indicate that the FTF-CIQ can be useful in this context. Due to the limited sensitivity of FTF-CIQ, it should not be used as the only screening instrument for decision of whether to perform a clinical assessment of NDD or not. Using the lower cut-off value (20.50) would miss 33% of true NDD cases and using the higher cut-off value (30.50) would still miss 23% of NDD cases. Further, the FTF-CIQ cut-offs cannot be used to discriminate between ASD and ADHD. Rather than screening for the specific diagnoses, the FTF-CIQ is a suitable instrument for rigorous, systematic assessment of broader developmental difficulties commonly associated and co-morbid with NDD:s. Due to the instability of categorical core symptoms over time [71] and large overlap between symptoms from several different NDD and psychiatric diagnostic categories, use of a broader instrument yielding a deeper phenotype characterisation beyond mere diagnostic criteria can aid the clinician in identifying the presence of developmental abnormalities in adults early in the assessment, and assist decisions of which diagnoses to focus on more narrowly.

### Limitations

The major limitation of the present study is that the control parents were recruited from a self-selected web panel. Putative selection biases cannot be identified since non-response analysis cannot be performed. A common limitation with web panels is that individuals who do not use internet are not randomly distributed and have a systematic bias for younger age and higher educational level. However, at the time when the general population data were collected, 93% of the Swedish population had access to internet and 91% actively used internet. In the age group > 46 years, approximately 2/3 of the population used internet for information searching. The e-mail invitation to participate in the study was sent to a stratified sample of individuals in the age range 45–50 years (25%) and > 50 years (75%), thus addressing the potential overrepresentation of younger respondents. The gender distribution of mothers and fathers completing the FTF in the original study by Kadesjöö et al. in children was closely replicated with 77% mothers and 23% fathers in our final sample, proportions that are in line with our clinical experience. Data on educational level for the respondents were not available, why our control group potentially could differ from the general population as well as from the clinical groups in this regard. Another limitation is the identification of absence of NDD in the control parents’ rating of their now adult offspring. Since no clinical assessment of the control group was performed, we cannot rule out the possibility of inclusion of undiagnosed NDD cases in the control group. This would result in an underestimation of the differences between control parents and parents to clinical cases, why the differences presented here should be viewed as a conservative estimate of the differences between non-NDD controls and NDD patients in retrospective parent FTF-CIQ rating.

Despite these limitations, our results indicate that FTF-CIQ in retrospective assessment of childhood NDD symptoms yields results comparable to those previously obtained for children, thus indicating a good face validity. Thus, FTF is an accessible (www.5-15.org) instrument that can enrich the assessment procedure of adult NDD:s in clinical practice by providing retrospective collateral information on childhood symptoms and difficulties.

## Electronic supplementary material

Below is the link to the electronic supplementary material.

Supplementary file1 (DOCX 28 kb)
