# Supplementary material for: Using the five to fifteen-collateral informant questionnaire for retrospective assessment of childhood symptoms in adults with and without autism or ADHD
Source: Eur Child Adolesc Psychiatry. 2020 Jul 25;30(9):1367–81. doi: 10.1007/s00787-020-01600-w (PMC8440248; doi:10.1007/s00787-020-01600-w)
Supplement: Supplementary file 1 — Supplementary file1 (DOCX 28 kb) [file 787_2020_1600_MOESM1_ESM.docx]

**Supplementary table A.** The items included in the Five to Fifteen domains and subdomains, as well as examples of items within each domain.

| **FTF-CIQ domains**  *subdomains* | **FTF Items included** | **Examples of items** |
| --- | --- | --- |
| **Motor skills** | 1-17 |  |
| *Gross motor skills* | 1-7 | Difficulty acquiring new motor skills, such as learning how to ride a bike, skate, swim |
| *Fine motor skills* | 8-17 | Often spills food onto clothes or table when eating |
| **Executive functions** | 18-42 |  |
| *Attention* | 18-26 | Often has difficulty sustaining attention in tasks or play activities |
| *Hyperactive/Impulsive* | 27-35 | In constant motion (squirms in seat, fidgets with fingers, plucks at things etc) |
| *Hypoactive* | 36-39 | Difficulty completing a task/activity, does not get things done like the rest of the group |
| *Planning/organizing* | 40-42 | Difficulty planning and preparing for tasks (e.g., collecting equipment needed for an outing or for school) |
| **Perception** | 43-60 |  |
| *Relation in space* | 43-47 | Bumps into other people, especially in narrow places |
| *Time concepts* | 48-51 | Repeatedly asks about when something is going to happen, e.g., how much time is left before an outing or before it is time to go to school |
| *Body perception* | 52-56 | Does not have a sense of how clothes fit, does not straighten socks or trousers that have slid down |
| *Visual perception* | 57-60 | Difficulty noticing small differences in shapes, figures, words and patterns that look alike |
| **Memory** | 61-71 | Difficulty remembering appointments with peers or what home-work he/she has got |
| **Comprehension** | 72-76 | Difficulty with abstract concepts such as “the day after tomorrow”, ”in the right order” |
| **Language** | 77-92 |  |
| *Expressive language skills* | 77-89 | Difficulty speaking fluently without any breaks |
| *Communication* | 90-92 | Difficulty taking part in a conversation, e.g., problems shifting from listening to talking |
| **Learning** | 93-121 (ecluding 110 and 111, assessing specific strenghts) |  |
| Reading/writing | 93-100 | Has difficulties to understand what he/she is reading |
| Math | 101-105 | Difficulty learning multiplication tables |
| General learning | 106-109 | Difficulty understanding or using abstract terms, e.g., terms relating to size, volume, spatial directions |
| Coping in learning | 112-121 | Difficulty comprehending explanations and following instructions given by adults |
| **Social skills** | 122-148 | Difficulty expressing emotions and reactions with facial gestures or body |
| **Emotional/ behavioural problems** | 149-181 |  |
| Internalising | 149-160 | Often expresses a feeling of being worthless or inferior to other children |
| Externalising | 161-173 | Often destroys the belongings of other family members or other children |
| Tics and obsessive-compulsive | 174-181 | Repeats meaningless movements, such as head shaking, body jerking and finger drumming |
